# Supplementary material for: Dysregulated nuclear Lamin B1 in DYT1 dystonia thickens nuclear lamina and disrupts 14-3-3 proteins
Source: Cell Death Discov. 2026 Apr 15;12:245. doi: 10.1038/s41420-026-03090-2 (PMC13195094; doi:10.1038/s41420-026-03090-2)
Supplement: Supplementary file 1 — Related Manuscript File [file 41420_2026_3090_MOESM1_ESM.pdf]

Supplemental Information for

Dysregulated nuclear Lamin B1 in DYT1 dystonia thickens the nuclear lamina and disrupts 14-3-3 proteins

Yuntian Duan[1], Masood Sepehrimanesh[1], Md Abir Hosain[1], Haochen Cui[1], Jacob Stagray[2], Xinggui Shen[3], Ying Xiao[4], Yuqing Li[5], Chun-Li Zhang[6] and Baojin Ding[1*]

[1]Department of Biochemistry and Molecular Biology, Louisiana State University Health Sciences Center at Shreveport, Shreveport, LA 71103, USA

[2]Department of Biology, University of Louisiana at Lafayette, Lafayette, LA 70503, USA

[3]Department of Pathology and Translational Pathobiology, Louisiana State University Health Sciences Center at Shreveport, Shreveport, LA 71103, USA

[4]Shared Instrumentation Facility, Louisiana State University, Baton Rouge, LA 70809, USA

[5]Norman Fixel Institute for Neurological Diseases, Department of Neurology, College of Medicine, University of Florida, Gainesville, FL 32610, USA

[6]Department of Molecular Biology, University of Texas Southwestern Medical Center, Dallas, TX 75390, USA

*Correspondence: Baojin.ding@lsuhs.edu

This PDF file includes:

Figures S1-S7

Tables S1-S9

Note: Supplemental tables S4-9 were uploaded separately as excel files to Figshare.

Supplementary Figures and Figure Legends

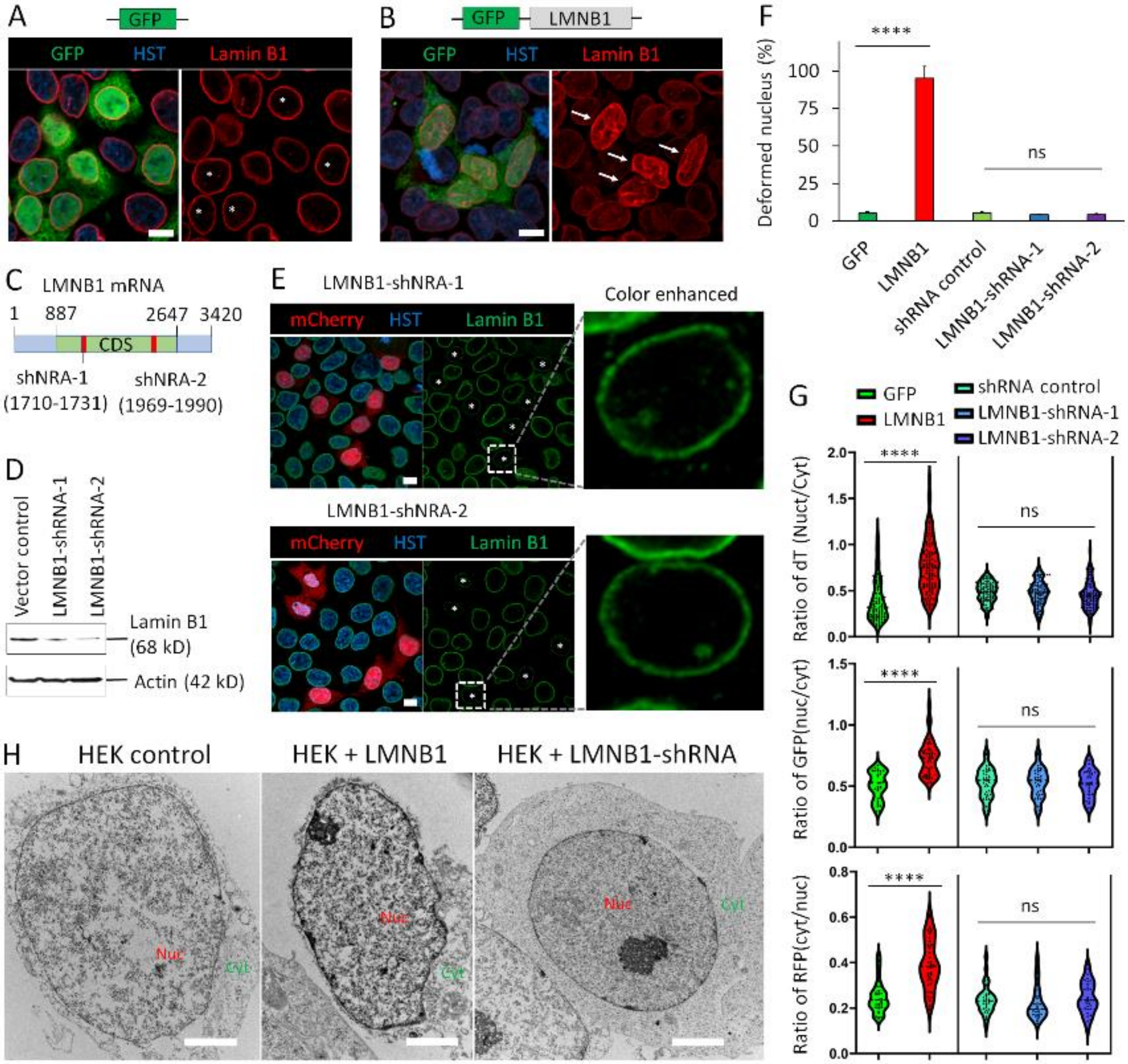

Figure S1. Upregulation of nuclear Lamin B1 significantly disrupts nuclear envelope morphology and nucleocytoplasmic transport, whereas downregulation does not.

(A) Representative confocal micrograph of human embryonic kidney (HEK) cells ectopically express GFP. Asterisks indicate typical nuclear morphology of GFP-positive cells. Scale bar: 10 μm.

(B) Representative confocal micrograph of HEK cells ectopically express nuclear Lamin B1 and the GFP reporter. Arrows indicate deformed nucleus with overexpression of Lamin B1. Scale bar: 10 μm.

(C) A schematic shows two shRNAs targeting different sites within the coding sequence (CDS) of the LMNB1 transcript.

(D) Western blot analysis shows that both LMNB1-targeting shRNAs efficiently down-regulate nuclear Lamin B1 protein levels. Beta-actin serves as a loading control.

(E) Representative confocal images of HEK cells expressing LMNB1-shRNAs. The transfected cells express mCherry and are marked with asterisks in Lamin B1 staining. Cells with white squares are also shown at higher magnification with enhanced contrast to highlight nuclear envelope morphology. Scale bars: 10 μm.

(F) Quantification of HEK cells with nuclear deformation under indicated conditions. n (cells) > 100 from three triplicates. ns, no significant difference; **** p<0.0001. Student's t-test.

(G) Quantification of nucleocytoplasmic transport under indicated conditions. FISH assay, n (cells) > 100 from triplicates. Reporter assay, n (cells) > 50 from triplicates. ns, no significant difference; **** p<0.0001. Student's t-test and one-way ANOVA.

(H) Representative transmission electron microscopy (TEM) images of HEK nuclei under indicated conditions. Nuc, nucleus; Cyt, cytoplasm. Scale bars: 2 μm.

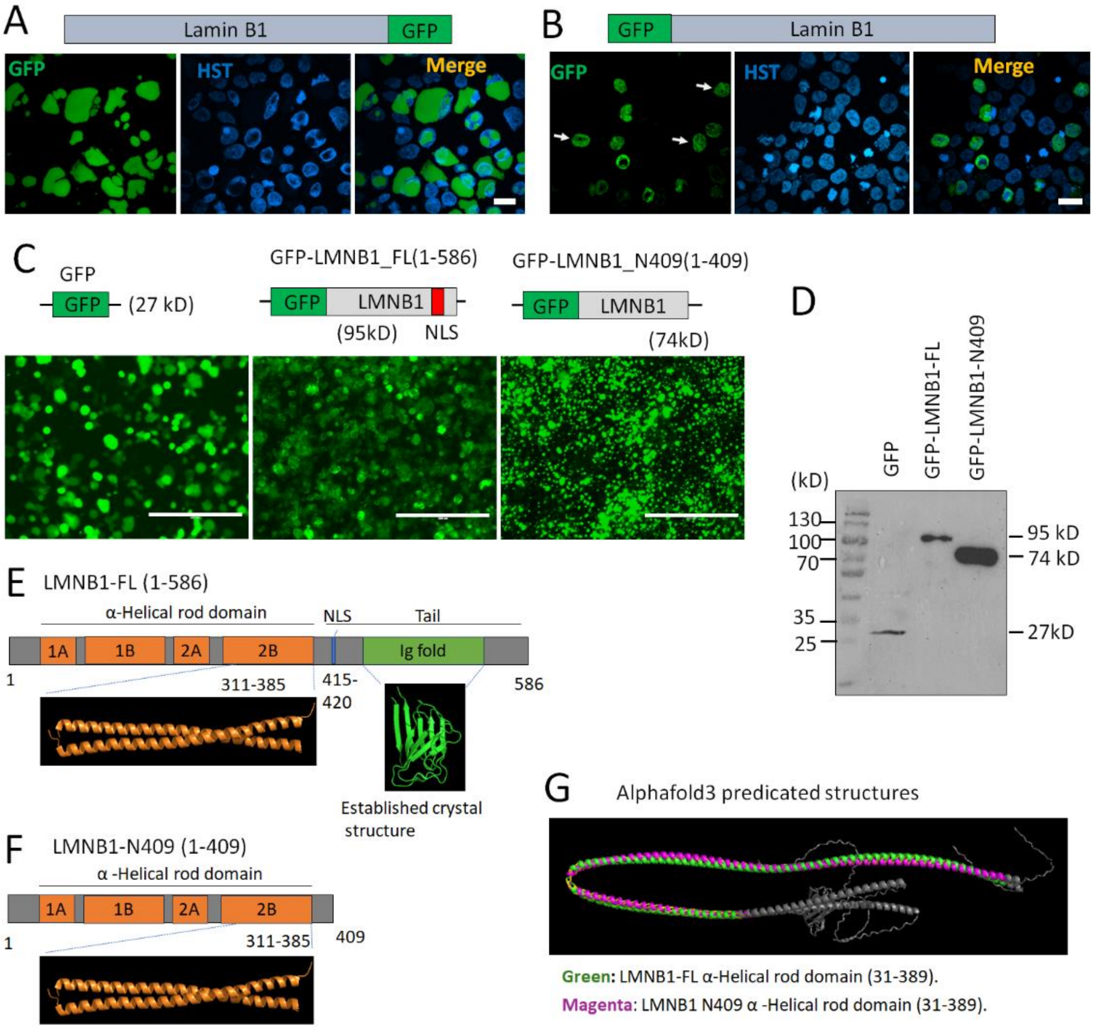

Figure S2. Overexpression of GFP-tagged full-length nuclear and truncated Lamin B1.
(A) Micrograph of HEK cells expressing full-length Lamin B1 with a C-terminal GFP tag. Scale bar: 20 µm.
(B) Micrograph of HEK cells expressing full-length Lamin B1 with an N-terminal GFP tag. Arrows indicate overexpressed LMNB1-GFP. Scale bar: 20 µm.
(C) Micrographs of HEK cells expressing GFP alone, N-terminally tagged full-length Lamin B1 (LMNB1_FL), or a truncated Lamin B1 lacking the nuclear localization signal (LMNB1_N409). Scale bars: 200 µm.
(D) Western blot showing expression of GFP, GFP tagged LMNB1_FL and LMNB1_N409, each at their expected molecular weight.
(E) Schematic of full-length LMNB1 protein domains, including regions with known crystal structures: the α-helical rod domain 2B and the Ig-fold domain in the C-terminal tail.
(F) Schematic of the LMNB1 truncation mutant (residues 1–409), highlighting the retained α-helical rod domain 2B and the absence of the C-terminal tail.
(G) AlphaFold3-predicted structural comparison of full-length LMNB1 and its truncation mutant.

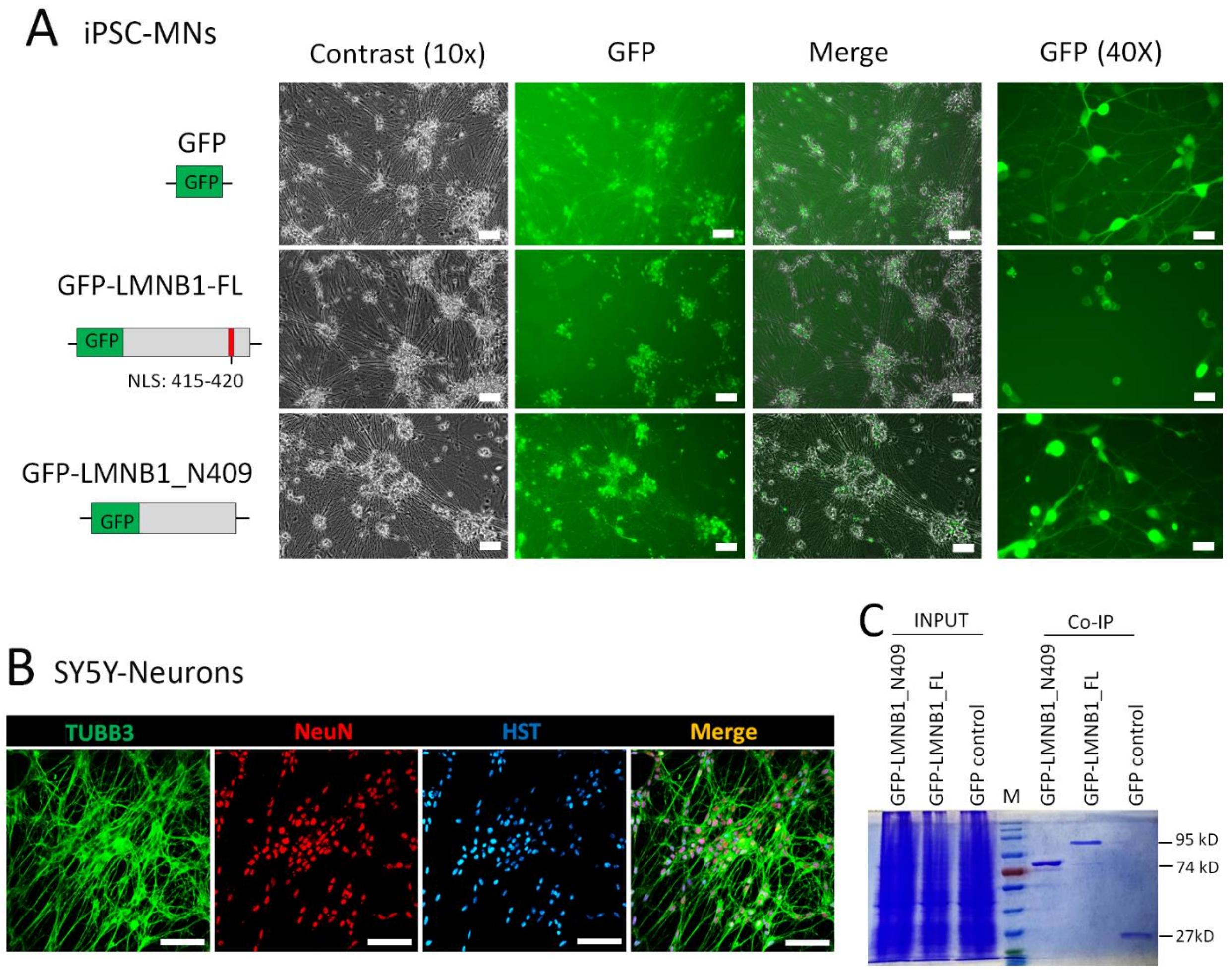

Figure S3. Overexpression of GFP-tagged Lamin B1 constructs in human induced neurons and immunoprecipitation sample preparation for proteomic analysis.
(A) Micrographs of hiPSC-derived motor neurons at 10 days post-infection (dpi) with lentiviruses expressing GFP, GFP-tagged LMNB1_FL, or LMNB1_N409. The truncation mutant lacks the NLS and mislocalizes to the cytoplasm and neuronal processes. Scale bars: 100 µm (10×), 20 µm (40×).
(B) Micrographs of neurons differentiated from the SH-SY5Y neuroblastoma cell line. Scale bar: 100 µm.
(C) Coomassie Brilliant Blue-stained SDS-PAGE gel of immunoprecipitated (IP) samples prepared from SH-SY5Y-derived neurons using anti-GFP nanobody-conjugated magnetic agarose beads. Each lane was loaded with either 1% of total cell lysate (INPUT) or 10% of the IP sample.

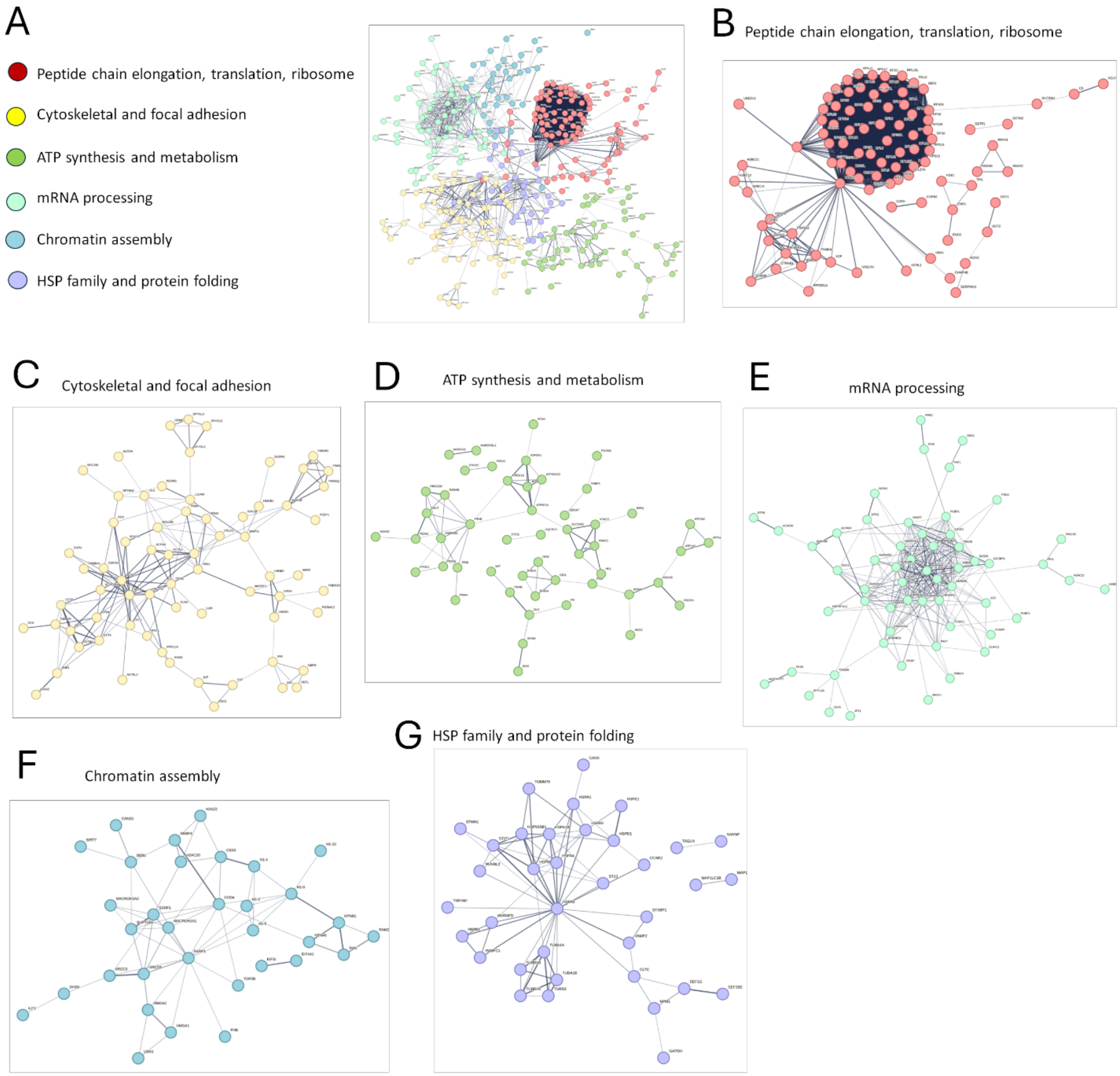

Figure S4. Lamin B1-interacting proteins in hiPSC-MNs are highly enriched in six clusters.
(A) Network analysis reveals that Lamin B1-interacting proteins form six distinct clusters based on functional enrichment.
(B–G) Detailed views of individual clusters:
(B) Peptide chain elongation, translation, and ribosome-related proteins.
(C) Cytoskeletal organization and focal adhesion components.
(D) ATP synthesis and metabolic processes.
(E) mRNA processing factors.
(F) Chromatin assembly-related proteins.
(G) Heat shock proteins (HSPs) and protein folding machinery.

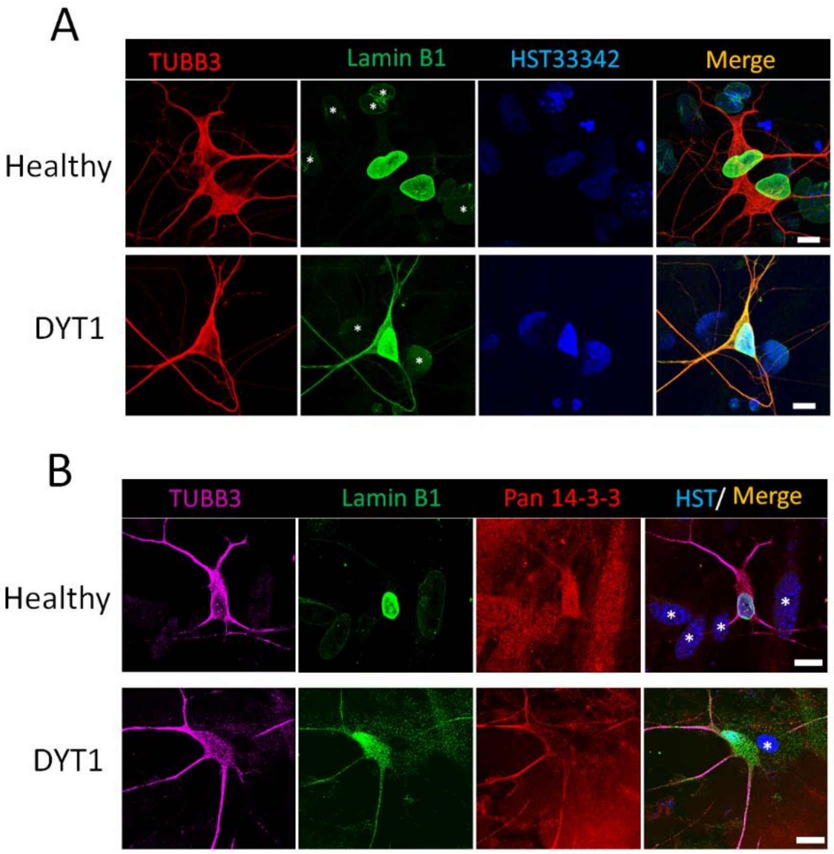

Figure S5. 14-3-3 proteins colocalize with cytoplasmically mislocalized Lamin B1 in DYT1 patient-derived motor neurons.

(A) Confocal micrographs of motor neurons directly reprogrammed from healthy control (GM00024) and DYT1 patient (GM03211) fibroblasts at 5 weeks post-viral infection (WPI). Asterisks indicate co-cultured astrocytes. Scale bars: 20 µm.

(B) Confocal micrographs showing co-staining of Lamin B1 and pan-14-3-3 proteins in reprogrammed neurons. Asterisks indicate co-cultured astrocytes. The pan-14-3-3 antibody, which recognizes all isotypes, stains both neurons and astrocytes, resulting in high background signal. Nevertheless, 14-3-3 proteins are enriched and colocalize with mislocalized cytoplasmic Lamin B1 in neuronal processes. Scale bars: 20 µm.

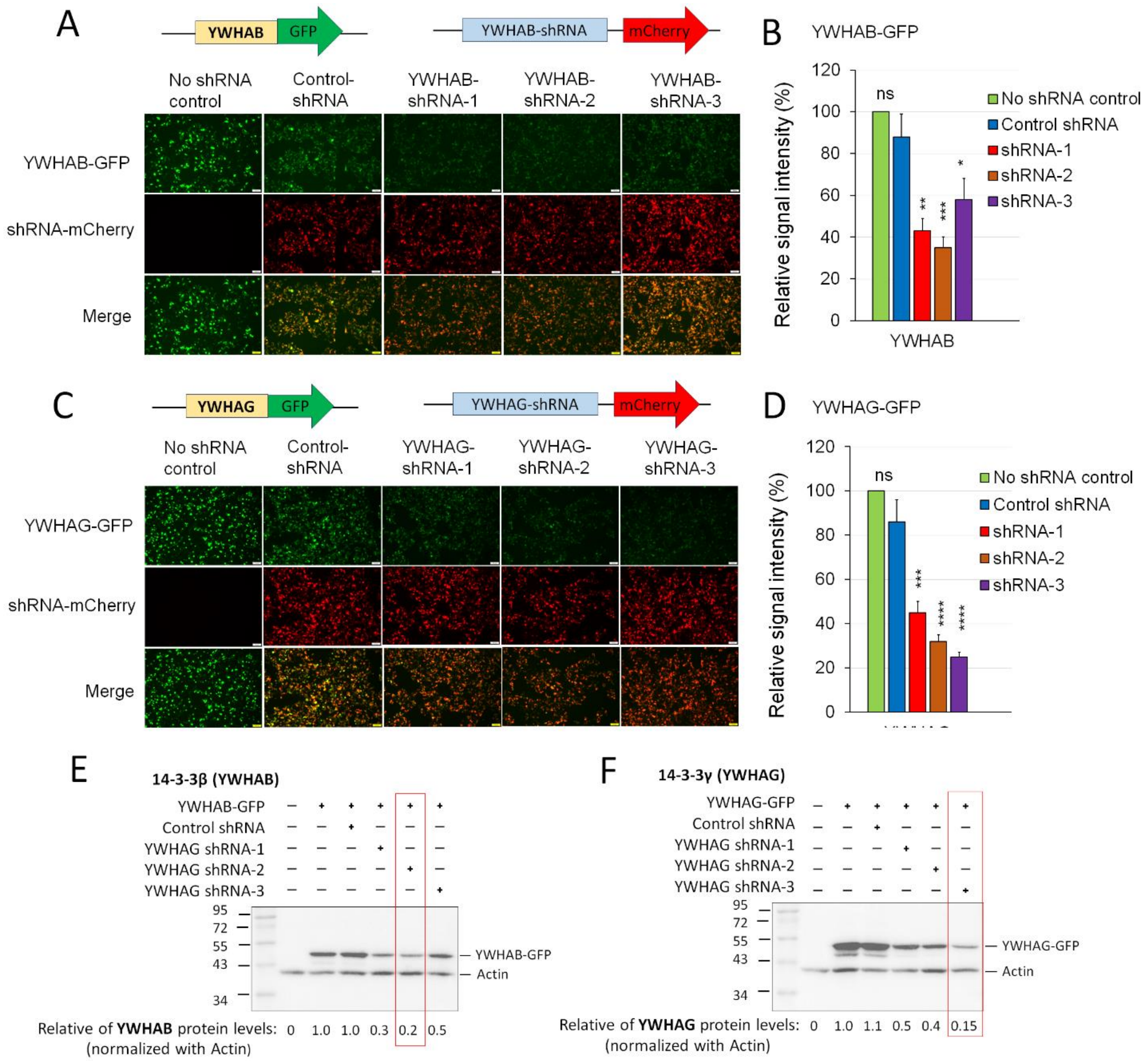

Figure S6. Design and validation of lentiviral vectors to modulate 14-3-3 expression.
(**A**) Fluorescence micrographs of HEK cells co-transfected with lentiviral vectors expressing GFP-tagged YWHAB and YWHAB-targeting shRNAs. Three shRNAs target different sites within the coding region of the YWHAB gene. A scrambled sequence was used as a control shRNA. Transfected cells are marked by mCherry, and GFP signal intensity reflects the relative expression level of YWHAB-GFP. Scale bar: 100 µm.
(**B**) Quantification of YWHAB-GFP fluorescence intensity from panel (A). Compared to control shRNA: ns, not significant; $*p < 0.05$; $**p < 0.01$; $***p < 0.001$. One-way ANOVA with Dunnett's multiple comparison test.
(**C**) Fluorescence micrographs of HEK cells co-transfected with GFP-tagged YWHAG and YWHAG-targeting shRNAs. Three shRNAs target different regions of the *YWHAG* coding sequence. Scale bar: 100 µm.
(D) Quantification of YWHAG-GFP fluorescence intensity from panel (C). Compared to control

shRNA: ns, not significant; ***p < 0.001; ****p < 0.0001. One-way ANOVA with Dunnett's multiple comparison test.

(**E**) Western blot showing YWHAB-GFP and β-actin protein levels from whole-cell lysates prepared from panel (A). Blots were sequentially probed with anti-GFP and anti-actin antibodies. Relative YWHAB-GFP protein levels are indicated below. YWHAB-shRNA-2 showed the strongest knockdown efficiency.

(F) Western blot showing YWHAG-GFP and β-actin protein levels from whole-cell lysates prepared from panel (C). Blots were sequentially probed with anti-GFP and anti-actin antibodies. Relative YWHAG-GFP protein levels are indicated below. YWHAG-shRNA-3 showed the strongest knockdown efficiency.

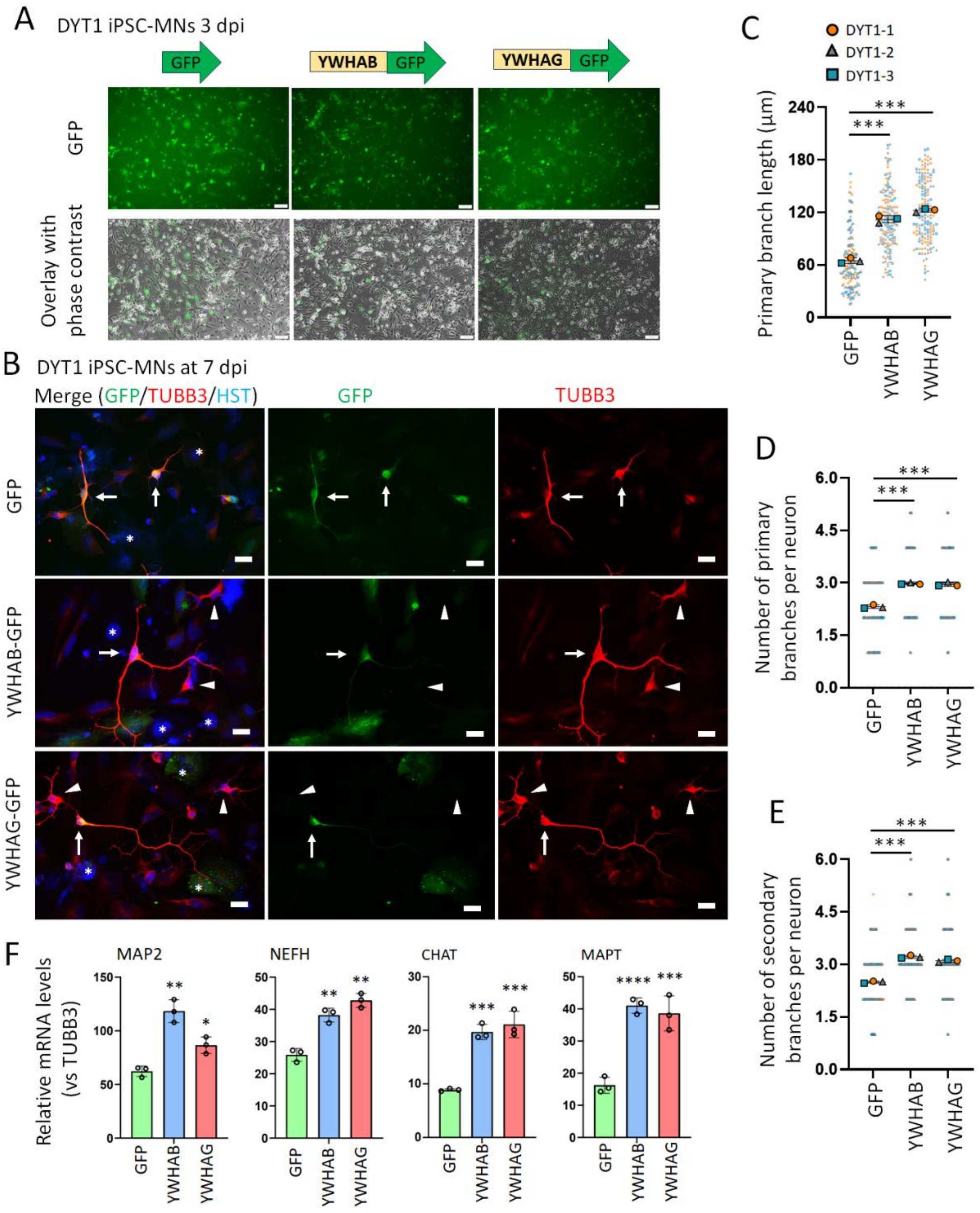

Figure S7. Overexpression of 14-3-3 proteins enhances neurite outgrowth and differentiation in DYT1 neurons.
(A) Representative micrographs of DYT1 iPSC-MNs (DYT1-1) transduced with lentiviruses expressing GFP, GFP-tagged YWHAB, or YWHAG at 3 dpi. Transduction efficiency and seeded neuron density are comparable across conditions. Scale bar: 100 μm.

(B) Fluorescence micrographs of DYT1 iPSC-MNs at 7 dpi. Arrows indicate GFP-positive (transduced) neurons; arrowheads indicate GFP-negative (non-transduced) neurons; asterisks denote co-cultured astrocytes. Scale bar: 20 µm.
(**C**) Quantification of relative neurite length in DYT1 iPSC-MNs at 7 dpi. N (objects) = 3. n (Neurons) = 50 for each object highlighted with distinct colors. Compared to control: *** $p<0.001$. One-way ANOVA with Dunnett's multiple comparison test.
(**D**) Quantification of primary neurite branches per neuron at 7 dpi. N (objects) = 3. n (Neurons) = 50 for each object highlighted with distinct colors. Compared to control: *** $p<0.001$. One-way ANOVA with Dunnett's multiple comparison test.
(**E**) Quantification of secondary neurite branches per neuron at 7 dpi. N (objects) = 3. n (Neurons) = 50 for each object highlighted with distinct colors. Compared to control: *** $p<0.001$. One-way ANOVA with Dunnett's multiple comparison test.
(**F**) Relative mRNA levels in DYT1-MNs at 7 dpi analyzed by RT-PCR. *n* = 3 biological replicates. Compared to control: * $p<0.05$, ** $p<0.01$, *** $p<0.001$, **** $p<0.0001$. One-way ANOVA with Dunnett's multiple comparison test.

Supplementary Tables

Table S1. List of cell lines used in this study and their Research Resource Identifiers (RRIDs).

	Resource	Sex	Age at Sampling (YR)	Tissue Type	Race	Onset Age (YR)	Gene	Mutation
**Fibroblast cell lines**								
DYT1-1	Coriell Cat# GM03211, RRID:CVCL_1U24	M	30	Skin	Caucasian	7	TOR1A	907_909delGAG
DYT1-2	NDS00305	M	22	Skin	Caucasian	12	TOR1A	907_909delGAG
DYT1-3	Coriell Cat# GM02304, RRID:CVCL_1U21	F	17	Skin	Caucasian	7	TOR1A	907_909delGAG
DYT1-4	NDS00301	F	53	Skin	Caucasian	9	TOR1A	907_909delGAG
Control-1	Coriell Cat# GM00024, RRID:CVCL_7269	M	31	Skin	Caucasian			
Control -2	Coriell Cat# GM03652, RRID:CVCL_7397	M	24	Skin	Caucasian			
Control -3	Coriell Cat# GM04506, RRID:CVCL_7413	F	20	Skin	Caucasian			
Control -4	Coriell Cat# AG07473, RRID:CVCL_2C33	F	50	Skin	Caucasian			
**hiPSC lines**								
DYT1-1	DYT1-1C8, Ref. (1)	M	30	TOR1A 907_909delGAG				
DYT1-2	DYT1-H6, Ref. (1)	M	30	TOR1A 907_909delGAG				
WT-1	WT-1A2, Ref. (1)	M	30	Isogenic control for 1C8 and H6				
WT-2	WT-1F6, Ref. (1)	M	30	Isogenic control for 1C8 and H6				
DYT1-3	DYT1-11, Ref. (2)	M	30	TOR1A 907_909delGAG				
WT-3	WT-4B2, Ref. (3)	M	30	Isogenic control for DYT1-11				
WT-4	WT-A6, Ref. (3)	M	30	Isogenic control for DYT1-11				
**HEK 293T cells**		ATCC Cat# CRL-11268, RRID:CVCL_1926						
**SH-SY5Y cells**		ATCC, Cat# CRL-11266, RRID:CVCL_0019						

Note: RRIDs, Research Resource Identifiers (RRIDs); M, Male; F, Female.

Table S2. Oligo Sequence

Name	Sequence (5'-3')	Note
LMNB1-shRNA-1	AGCTTCTTGATGTAAAGTTA	gRNA
LMNB1-shRNA-2	CAGACTGTCATCAGAGATGAA	gRNA
YWHAB-shRNA-1	GAAGGTTTAATCTGATATCAAA	gRNA
YWHAB-shRNA-2	TAAAGTTGTACAGAAAGTTATA	gRNA
YWHAB-shRNA-3	AACCAGAAAGTAAGGTGTTCTA	gRNA
YWHAG-shRNA-1	ACAGACTCAATGTGCTCTGTAA	gRNA
YWHAG-shRNA-2	CCAGGTTGGTTTTGCTGTTGAT	gRNA
YWHAG-shRNA-3	GTCCAAGGTTTTCTATTATAAA	gRNA
TUBB3-F	CACCCAGCAGATGTTCGATG	RT-PCR
TUBB3-R	CTGTTCTTGCTCTGGATGGC	RT-PCR
MAP2-F	ACTCCTGGAACCCCTAGCTA	RT-PCR
MAP2-R	TGGGAGTCGCAGGAGATTTT	RT-PCR
NEFH-F	TGAGCTGAGGAACACCAAGT	RT-PCR
NEFH-R	AGCCAATCCGACACTCTTCA	RT-PCR
CHAT-F	ACAACCACGGAGATGTTCTG	RT-PCR
CHAT-R	TGCAGCTGTGAAAGCTAGAG	RT-PCR
MAPT-F	CAAAGCTCGCATGGTCAGTA	RT-PCR
MAPT-R	AGGGTTGGATCAGAGGGTCT	RT-PCR
YWHAB-F	TTCCCAATGCTACACAACCA	RT-PCR
YWHAB-R	AGTGCCAGACCAAGACGAAT	RT-PCR
YWHAG-F	CTGAATGAGCCACTGTCGAA	RT-PCR
YWHAG-R	GCACGGACCATCTCAATCTT	RT-PCR
YWHAE-F	ACTGGCGAGTCCAAGGTTTT	RT-PCR
YWHAE-R	TGCGTTGGTGGAAGTTCTGT	RT-PCR
YWHAH-F	GACATGGCCTCCGCTATGAA	RT-PCR
YWHAH-R	CCTGGCACCAACCACATTCT	RT-PCR
YWHAQ-F	GGAGAAAGTGGAGTCCGAGC	RT-PCR
YWHAQ-R	CGATCATCACCACACGCAAC	RT-PCR
YWHAZ-F	TTCTTGATCCCCAATGCTTC	RT-PCR
YWHAZ-R	AGTTAAGGGCCAGACCCAGT	RT-PCR
YWHAS-F	CATCATTGACTCAGCCCGGT	RT-PCR
YWHAS-R	TGTTGGCGATCTCGTAGTGG	RT-PCR

Table S3. List of primary antibodies used this study.

Name	Source	Catalogue	Host	Immunostaining	Western Blot
Pan 14-3-3	Santa Cruz	sc-1657	Ms	1:100	1:250
DIG	Sigma	11 333 089 001	Sh	1:100	-
GFP	Aves	GFP-1020	Ck	1:1000	-
LMNA/C	Abcam	ab40567	Ms	1:300	1:1000
LMNB1	PeproTech	12987-1-AP	Rb	1:500	1:2000
MAP2	Abcam	Ab5392	Ck	1:10000	-
NeuN	Abcam	Ab177487	Rb	1:100	-
TOR1A	Cell Signaling	2150	Ms	-	1:1000
TUBB3	Covance	MMS-435P	Ms	1:2000	-
TUBB3	Covance	PRB-435P	Rb	1:2000	-
β-Actin	Sigma	A5441	Ms	-	1:5000

Note: Ck, chicken; Gt, goat; Ms, mouse; Rb, rabbit; Sh, sheep; - not determined in this study.

Table S4. Co-IP/MS dataset from hiPSC-MNs and the list of reported Lamin B1-interacting proteins.

Table S5. GO analysis dataset of Lamin B1-interacting proteins in hiPSC-MNs.

Table S6. List of nuclear Lamin B1-associated proteins involved in regulating nuclear transport in iPSC-MNs.

Table S7. Categories of neuron-specific proteins associated with nuclear Lamin B1 in iPSC-MNs.

Table S8. List of proteins significantly changed the interaction with cytoplasmic Lamin B1 in iPSC-MNs

Table S9. List of proteins significantly changed the interaction with cytoplasmic Lamin B1 in SY5Y-Neurons.

Note: Supplemental tables S4-9 were uploaded separately as excel files.

1. M. Akter, H. Cui, Y. H. Chen, B. Ding, Generation of two induced pluripotent stem cell lines with heterozygous and homozygous GAG deletion in TOR1A gene from a healthy hiPSC line. *Stem Cell Res* **56**, 102536 (2021).
2. B. Ding *et al.*, Disease Modeling with Human Neurons Reveals LMNB1 Dysregulation Underlying DYT1 Dystonia. *J Neurosci* **41**, 2024-2038 (2021).
3. M. Akter, H. Cui, Y. H. Chen, B. Ding, Generation of gene-corrected isogenic control cell lines from a DYT1 dystonia patient iPSC line carrying a heterozygous GAG mutation in TOR1A gene. *Stem Cell Res* **62**, 102807 (2022).
