# Supplementary material for: Dysregulated nuclear Lamin B1 in DYT1 dystonia thickens nuclear lamina and disrupts 14-3-3 proteins
Source: Cell Death Discov. 2026 Apr 15;12:245. doi: 10.1038/s41420-026-03090-2 (PMC13195094; doi:10.1038/s41420-026-03090-2)

Supplemental Material: full and uncropped western blots.

Figure 1F: Western blot showing TorsinA protein levels in healthy and DYT1 fibroblasts.

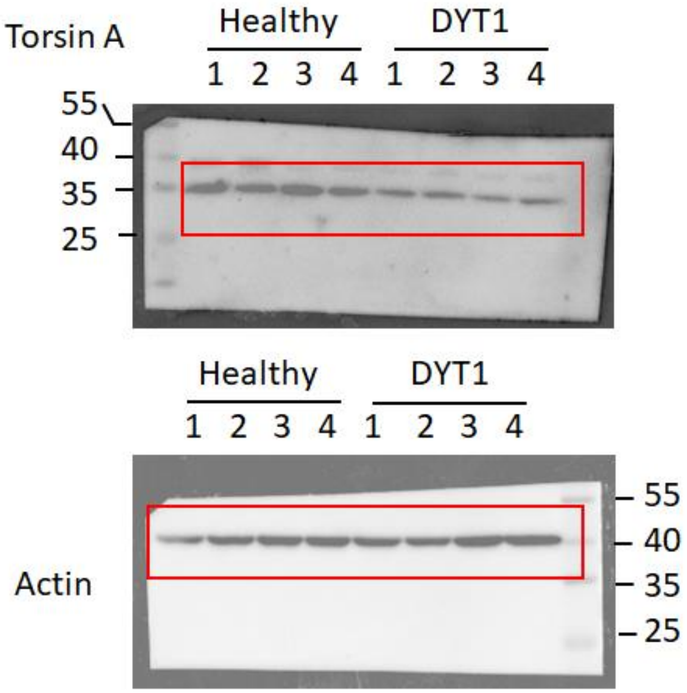

Figure 1H: Western blots showing protein levels of nuclear Lamin B1 and Lamin A/C in healthy and DYT1 fibroblasts.

Lamin A/C and Actin on the same blot:

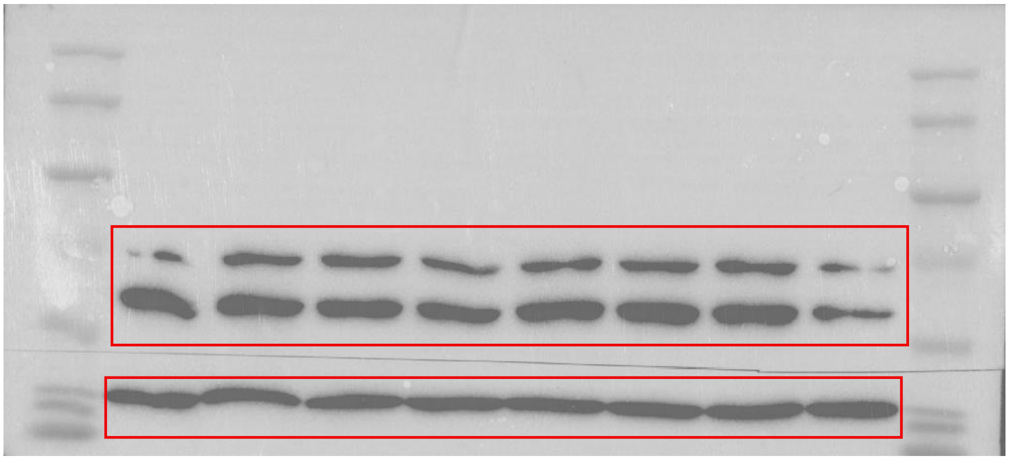

Lamin B1 on another blot with the same order and amount of loading.

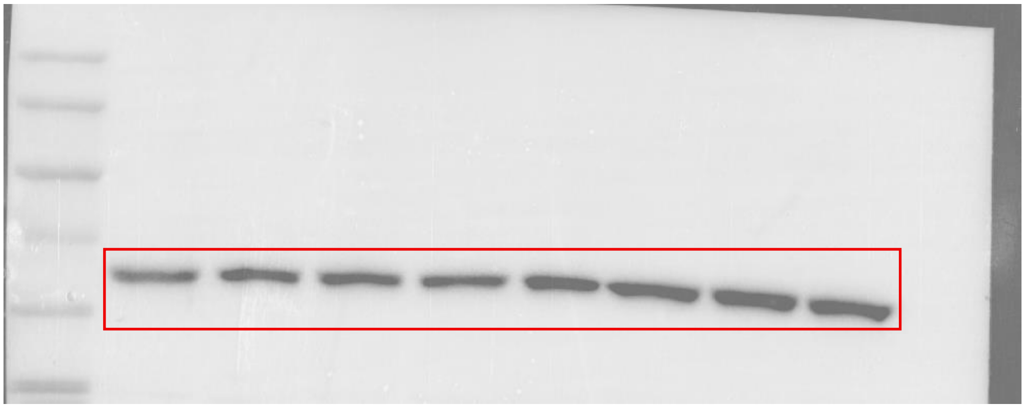

Figure 1J: Western blot analysis of nuclear and cytoplasmic fractions of Lamin B1 in healthy and DYT1 fibroblasts.

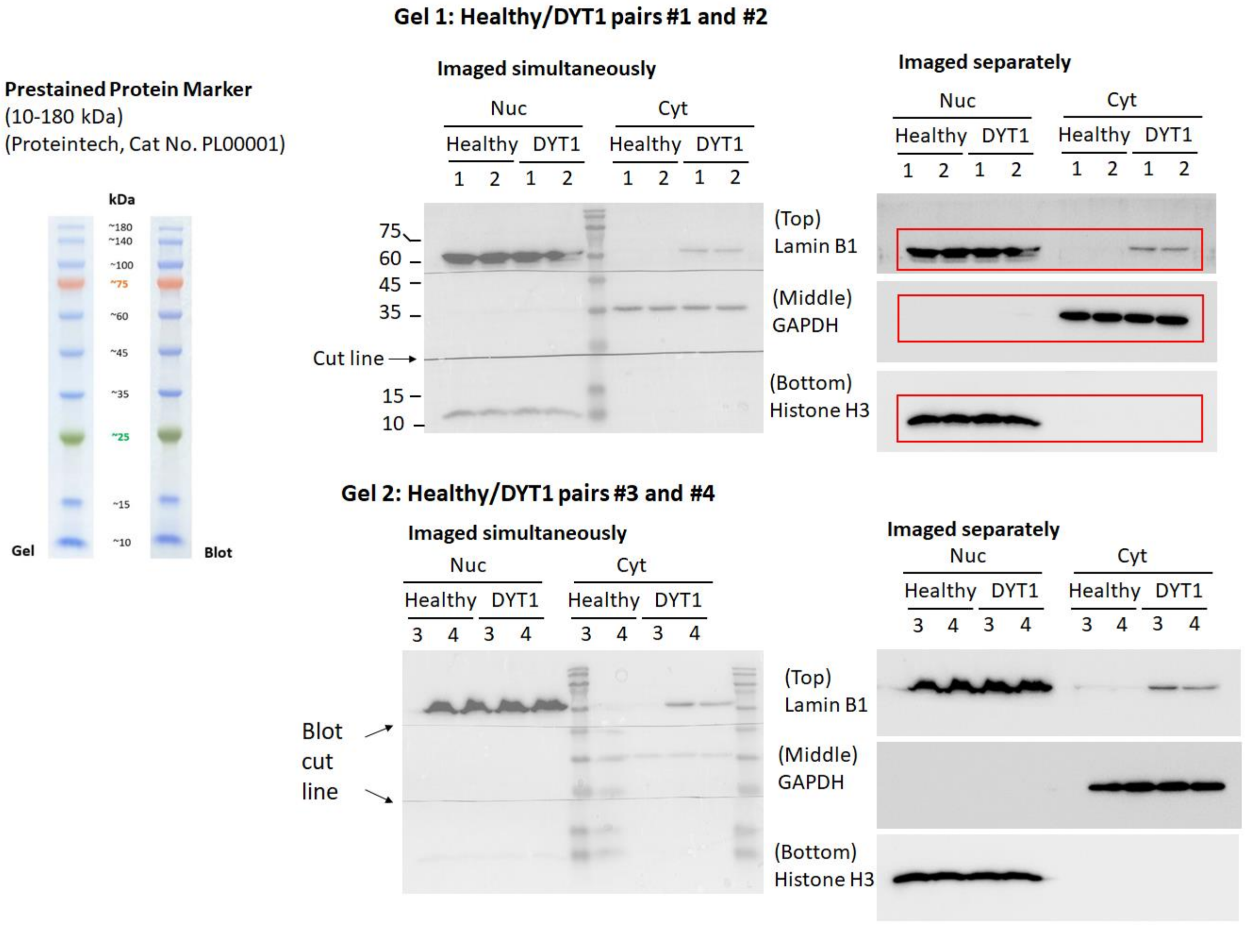

Figure 5I: Western blot validation of LMNB1 interactors. Input samples (2%) and Co-IP eluates from hiPSC-MNs expressing GFP, LMNB1_FL-GFP, or LMNB1_N409-GFP were probed using anti-GFP and pan–14-3-3 antibodies. M: protein marker.

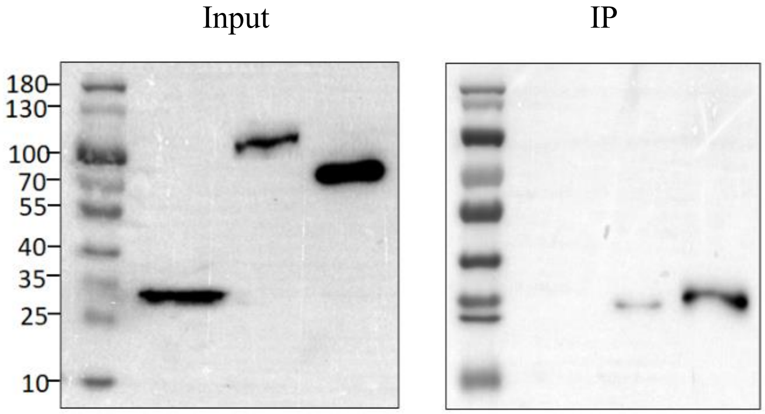

Figure S2D: Western blot showing expression of GFP, GFP tagged LMNB1_FL and LMNB1_N409, each at their expected molecular weight.

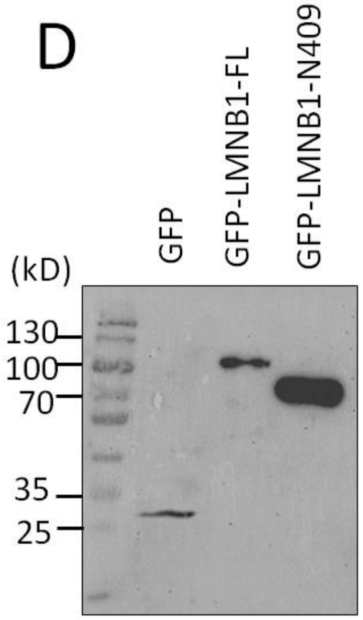

Figure S6E: shRNA-YWHAB

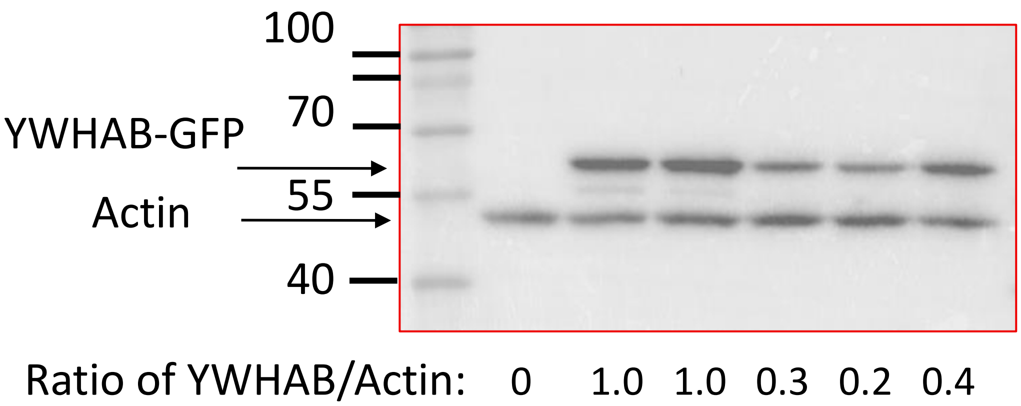

Figure S6F: shRNA-YWHAG

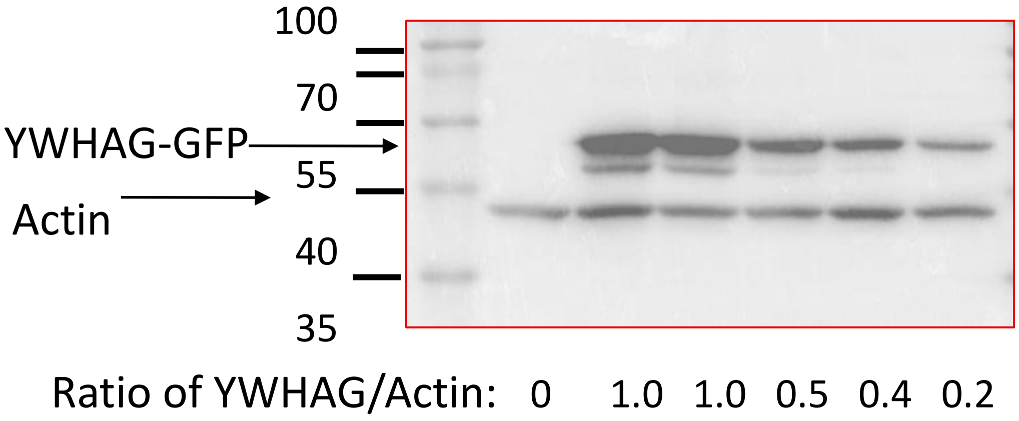

Supplement: Supplementary file 2 — Supplementary Material_uncropped WB [file 41420_2026_3090_MOESM2_ESM.pdf]
